# Idiopathic Gingival Hyperplasia

**Published:** 2009-06

**Authors:** Ferhat Cekmez, Ozgur Pirgon, Ilhan Asya Tanju

**Affiliations:** *Department of Pediatrics, GATA Medical Faculty, Istanbul, Turkey*

**Keywords:** gingival, hyperplasia, fibromatosis

## Abstract

Gingival hyperplasia is a rare condition but it is important for cosmetic and mechanic reasons and because of its potential as an indicator of systemic disease. Gingival fibromatosis may exist as an isolated abnormality or as part of a syndrome. In this article a case that was diagnosed clinically and histologically as idiopathic gingival fibromatosis is presented. Patient with gingival hyperplasia should be examined to exclude other reasons to determine the idiopathic gingival fibromatosis or not. Treatment is not required in all cases of idiopathic gingival hyperplasia. Surgical excision is indicated if mechanical problems exist. Recurrence has not been reported.

## INTRODUCTION

Gingival hyperplasia is a rare condition but it is important for cosmetic and mechanic reasons or possibility of a part of a systemic disease. In some pathological conditions, gingivitis caused by plaque accumulation can be more severe. In puberty and pregnancy, hyperplasia of the gingival tissues may be due to poor oral hygiene, inadequate nutrition, or systemic hormonal stimulation (1, 2). Gingival enlargements are also seen in several blood dyscrasias e.g. leukaemia, thrombocytopenia, or thrombocytopathy (3). Other etyologic factors are listed in Table 1. A progressive fibrous enlargement of the gingiva is a feature of idiopathic fibrous hyperplasia of the gingiva. Characteristically, this massive enlargement appears to cover the tooth surfaces. While the cause of the disease is unknown, there appears to be a genetic predisposition (4, 5). Gingival fibromatosis may exist as an isolated abnormality or as part of a syndrome (6, 7). Table 2 gives an overview of syndrome related gingival overgrowth. In this article, a 12 year girl who applied to pediatric service with the gingival hyperplasia is presented.

**Table 1:** Causes of gingival hyperplasia

Visuals aspect	Cause

Gingivitis	Bacterial plaque
More severe gingivitis diabetes	Bacterial plaque and uncontrolled
Puberty or pregnancy epulides	Bacterial plaque and puberty or pregnancy
Drug-induced gingival over-growth phenytoin, Dilantin	Bacterial plaque and medicine
Enlarged, oedematous, soft and tender, easily bleeding gingivitis	Leukaemia
Gingival enlargement and spontaneous bleeding	Thrombocytopenia and thrombocytopathy
Part of a syndrome	See Table 2

**Table 2:** An overview of gingival overgrowth related with a syndrome

Syndrome	Symptoms other than gingival overgrowth	Heredity

Rutherfurd Syndrome	Corneal dystrophy	Dominant
Cross Syndrome	Microphthalmia, mental retardation, pigmentary defects	Recessive
Ramon Syndrome	Hypertrichosis, mental retardation, delayed development epilepsy, cherubism	Recessive
Laband Syndrome	Syndactily, nose and ear abnormalities, hyperplasia of the nails and terminal phalanges	Dominant

## CASE REPORT

**Figure 1:**
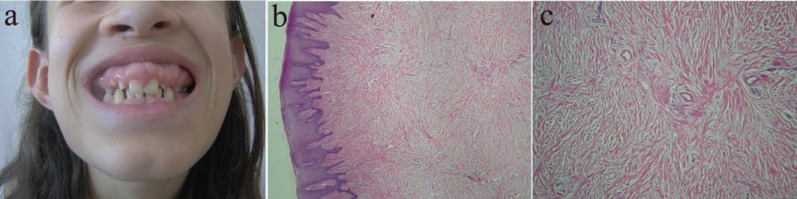


Patient has had gingival problems for 5 years. There were no any systemic diseases and drug using reported. In intraoral examination, the hyperplastic gingiva covered the teeth. Especially at the palatinal region this hyperplasia covered the palatinal dome and the tongue movements were restricted and speech trouble was seen. The gingival hyperplasia presented with colour. Complete blood cell count and chemistry tests, urinary and blood aminoacids, mucopolysccarides and hormonal profiles were normal. With the clinical and the histopathological examinations, the case was diagnoised as idiopatic gingival fibromatosis which was characteristed by fibrous gingival hyperplasia (Figure 1).

## DISCUSSION

Gingival fibromatosis may exist as an isolated abnormality or as part of a syndrome (6, 7). As an isolated finding, it is mostly sporadic, but an autosomal dominant inheritance pattern is also possible. Rarely, autosomal recessive inheritance is found.

Patients with gingival hyperplasia should be examined carefully and blood samples sould be taken to exclude blood dyscrasias (3). While the gingiva may be the only tissue involved, some cases display gingival fibromatosis in association with hypertrichosis, and/or mental retardation, and/or epilepsy. The association of gingival fibromatosis and corneal dystrophy is recognized as an autosomal dominant trait known as the Rutherfurd syndrome (6). Cross syndrome is, almost certainly, an autosomal recessive disorder characterized by gingival fibromatosis, microphthalmia, mental retardation, and pigmentary defects (7). Ramon syndrome is another, probably autosomal recessive, condition involving gingival fibromatosis, as well as hypertrichosis, mental retardation, delayed development, epilepsy and cherubism (8). Laband syndrome features gingival fibromatosis, syndactily, nose and ear abnormalities, and hypoplasia of the nails and terminal phalanges.

After excluding other reasons of gingival hyperplasia it is named as idiopathic gingival hyperplasia. Treatment is not required in all cases of idiopathic gingival hyperplasia. Surgical excision is indicated if mechanical problems exist (9). Recurrence has not been reported.
